# Retracted: Progesterone and Src Family Inhibitor PP1 Synergistically Inhibit Cell Migration and Invasion of Human Basal Phenotype Breast Cancer Cells

**DOI:** 10.1155/2022/9810368

**Published:** 2022-04-08

**Authors:** BioMed Research International

*BioMed Research International* has retracted the article titled “Progesterone and Src Family Inhibitor PP1 Synergistically Inhibit Cell Migration and Invasion of Human Basal Phenotype Breast Cancer Cells” [1] due to concerns with images as follows:

- Panels C to PP1+4 for rows MB231 and MB231Br in Figure 3 appear to be duplicated but distorted in Figure 5 as Panels C to PP1+4 for rows 231 and 231br
- Panels C and PP1 for MB231br in Figure 4 appear to be overlapping
- Panels C and P4 for MB231 in Figure 4 appear to be overlapping

As Figures 3, 4 and 5 show the migration of BPBC cell lines (specifically MB231Br for the latter two figures) under different treatments, no overlap is expected between the figures.

The authors responded to explain that the incorrect images were included due to a mistake when preparing the manuscript, however this did not satisfy the concerns of the Editorial Board, and the article is therefore being retracted due to concerns with the reliability of the data. The authors do not agree to the retraction.
